# Estimating the prevalence of illicit opioid use in New York City using multiple data sources

**DOI:** 10.1186/1471-2458-12-443

**Published:** 2012-06-18

**Authors:** Jennifer McNeely, Marc N Gourevitch, Denise Paone, Sharmila Shah, Shana Wright, Daliah Heller

**Affiliations:** 1 Department of Population Health, NYU School of Medicine, New York, NY, 10016, USA; 2 Division of General Internal Medicine, Department of Medicine, NYU School of Medicine, New York, NY, 10016, USA; 3 Bureau of Alcohol and Drug Use Prevention, Care and Treatment, New York City Department of Health and Mental Hygiene, Long Island City, NY, 11101, USA; 4 Department of Psychiatry, NYU School of Medicine, New York, NY, 10016, USA; 5 Center for Health Media and Policy, Hunter-Bellevue School of Nursing, City University of New York, New York, NY, 10010, USA

**Keywords:** Substance use, Substance abuse, Prevalence, Opioids, Heroin, Epidemiology

## Abstract

****Background**:**

Despite concerns about its health and social consequences, little is known about the prevalence of illicit opioid use in New York City. Individuals who misuse heroin and prescription opioids are known to bear a disproportionate burden of morbidity and mortality. Service providers and public health authorities are challenged to provide appropriate interventions in the absence of basic knowledge about the size and characteristics of this population. While illicit drug users are underrepresented in population-based surveys, they may be identified in multiple administrative data sources.

****Methods**:**

We analyzed large datasets tracking hospital inpatient and emergency room admissions as well as drug treatment and detoxification services utilization. These were applied in combination with findings from a large general population survey and administrative records tracking prescriptions, drug overdose deaths, and correctional health services, to estimate the prevalence of heroin and non-medical prescription opioid use among New York City residents in 2006. These data were further applied to a descriptive analysis of opioid users entering drug treatment and hospital-based medical care.

****Results**:**

These data sources identified 126,681 cases of opioid use among New York City residents in 2006. After applying adjustment scenarios to account for potential overlap between data sources, we estimated over 92,000 individual opioid users. By contrast, just 21,600 opioid users initiated drug treatment in 2006. Opioid users represented 4 % of all individuals hospitalized, and over 44,000 hospitalizations during the calendar year.

****Conclusions**:**

Our findings suggest that innovative approaches are needed to provide adequate services to this sizeable population of opioid users. Given the observed high rates of hospital services utilization, greater integration of drug services into medical settings could be one component of an effective approach to expanding both the scope and reach of health interventions for this population.

## **Background**

While there is great concern about the impact of illicit drug use in New York City, particularly use of heroin and prescription opioids, since the 1970s there have been few rigorous efforts to estimate its prevalence [1]. In recent decades we have seen significant changes in the culture and patterns of drug use, including greater availability of potent prescription opioids and decreasing rates of intravenous drug use. What persists is the disproportionate burden of morbidity and mortality borne by opioid users, including chronic infectious diseases (hepatitis C, HIV/AIDS) and premature death due to overdose and accidents [2-7]. Service providers and government health authorities are challenged to provide appropriate drug services in the absence of basic knowledge about the size and characteristics of their target population.

Three notable developments in recent years make this an opportune time to revisit estimates of the prevalence of opioid use. First, increasing rates of prescription opioid misuse and related overdose deaths indicate a shift in drug use patterns, demographics, and potential harms of use [8-11]. Prescription opioid users may be poorly reached by traditional drug treatment services, although treatment admissions in New York State do display a steady increase from this group over the past 15 years. Historically, research on opioid use has largely focused on heroin-using populations, with much less known about the treatment needs and access to care issues experienced by prescription opioid users [12].

A second event is the approval in 2002 of buprenorphine for treatment of opioid dependence, which offers an exceptional opportunity for expansion of evidence-based treatment. Buprenorphine can be prescribed by physicians in general practice settings, and thus offers the possibility of dramatically expanding access to treatment, but is not currently being employed to its fullest potential [13,14]. A related third event is a newly increased emphasis on integrating addiction treatment into non-specialty medical settings such as primary care clinics. This is exemplified in the current strategic plan issued by the federal Office of National Drug Control Policy, which emphasizes early identification of substance use disorders and expansion of treatment through mainstream healthcare settings [15].

Population surveys, primarily the National Survey on Drug Use and Health (NSDUH), are relied upon to assess the prevalence of illicit drug use, but are believed to severely underestimate ‘hardcore’ drug use, including heroin and injection drug use [16,17]. The survey does not include individuals who are incarcerated, institutionalized, or street homeless; all populations with disproportionately high prevalence of drug use [18]. It may also underestimate drug use among those who do participate, due to underreporting of stigmatized and illicit behavior by respondents, despite efforts to counteract this with recent changes in survey methodology [19]. As a result of these constraints, the NSDUH is limited in its ability to inform intelligent program design for opioid users.

Other data collected for administrative purposes and public health surveillance contains information on the health, demographic, and geographic characteristics of opioid users, and captures populations excluded from the NSDUH sample. While no single data source provides a comprehensive picture of illicit opioid use, each provides information on a subset of users who have developed social, psychological or physical problems as a consequence of their use. Yet these data sources have not previously been systematically applied to characterize the prevalence of opioid use. We analyzed these data with a goal of informing potential changes in drug services that could accommodate those left out of the current treatment system.

## **Methods**

This descriptive analysis of the prevalence of opioid use in New York City utilizes multiple data sources, including administrative datasets recording hospital inpatient and emergency room admissions, treatment and detoxification services utilization, prescription records, overdose deaths, and correctional health statistics, as well as survey data from the NSDUH. Using data from the most recent year (2006) for which complete data were available for all sources, we analyzed these datasets in combination to characterize the population of opioid users who would be candidates for drug services. We then compared them to the population entering drug treatment programs to estimate the extent of the untreated opioid user population.

### *Definition of opioid use/users*

Opioid users are defined as individuals using heroin or misusing prescription opioids (taking prescription opioid medications outside of a physician’s care or other than as prescribed). Opioid use is potentially, but not necessarily, concurrent with the clinical diagnoses of opioid abuse or dependence. While not all datasets allow a rigorous classification of abuse or dependence, opioid users are identified in the data by virtue of the health or treatment consequences of their opioid abuse, dependence, or poisoning, and are thus all potential candidates for drug services [20,21].

### *Case definition*

Cases were limited to unique individuals because our goal was to identify the size and characteristics of the opioid user population rather than service utilization. Cases were additionally limited to residents of the five counties of New York City. Specific definitions for identifying cases of opioid use varied by dataset, and are described in Table 1.

**Table 1:** Description of Datasets and Case Definitions

**Dataset**	**Source**	**Population Type Captured**	**Information Collected**	**Case Definition**	**Year**
**PRIMARY DATASETS**					
Statewide Planning and Research Cooperative System (SPARCS)	New York State Dept. of Health	1. Individuals discharged from hospital (inpatient dataset) 2. Individuals admitted to emergency room (outpatient dataset)	· Demographic characteristics · Zip code of residence · Procedures and principal, secondary and admitting diagnoses, classified by ICD-9 codes^i^ · Services received · Charges for treatment	· Age > 18 years · Zip code of residence within New York City · Hospital located in New York City · Opioid use as principal or secondary diagnosis, based on ICD-9 codes^ii^ · Unique individual, identified by SPARCS unique identifier + date of birth + sex · No detoxification or rehabilitation admissions	2006
Client Data System (CDS)	New York State Dept. of Health, Office of Alcohol and Substance Abuse Services	1. Individuals admitted to inpatient medical detoxification (crisis admissions) 2. Individuals admitted to New York State licensed drug treatment programs, including methadone maintenance	· Demographic characteristics · Zip code of residence · · Substance use behaviors, based on client self-report (includes current drugs of abuse, frequency of use and mode of administration)	· Age > 18 years · Zip code of residence within New York City · Opioid drug^iii^ reported as drug of abuse (primary, secondary, or tertiary drug) in at least one admission · Unique individual, identified by unique identifier constructed from sex + date of birth + last four digits of social security number + first two characters of last name · First admission of calendar year	2006
**SUPPLEMENTAL DATASETS**					
National Survey on Drug Use and Health (NSDUH)	U.S. Department of Health and Human Services, Substance Abuse and Mental Health Services Administration	General civilian population - Excludes institutionalized persons - Includes residents of noninstitutional group quarters (e.g. shelters, rooming houses)	· No individual identifying information · Region of residence · Substance use behaviors, based on self-report (includes substances used; lifetime, past year and current drug use; frequency and severity of use)	· Age > 12 years · Surveyed in New York City (New York Substate Region A)^iv^ · Opioid abuse or dependence, classified using DSM-IV criteria as per standard NSDUH methodology^v^	2005-2006 (avg)^vi^
Correctional Health Services program statistics	Correctional Health Services, New York City Dept. of Health and Mental Hygiene	Jail inmates - Serving sentence of one year or less, or - Detainee facing potential sentence of one year or less	· Number of inmates receiving methadone for opioid dependence (detoxification or maintenance treatment)	· Age > 18 years · In New York City jail facility providing methadone for opioid dependence^vii^ · Receiving methadone for detoxification or maintenance treatment	2006
Vital Statistics	Bureau of Vital Statistics, and Bureau of Alcohol and Drug Use Prevention, Care and Treatment of New York City Dept. of Health and Mental Hygiene	Drug overdose decedents	· Unintentional drug overdose deaths, based on Medical Examiner records^viii^	· All ages · Death occurred in NYC · Unintentional drug overdose deaths as defined by NYC DOHMH, based on manner of death and underlying cause of death recorded on death certificates	2006
Medication prescription records	New York City Dept. of Health and Mental Hygiene	Individuals initiating buprenorphine treatment	· Number of buprenorphine prescriptions filled each month at NYC pharmacies · Number of buprenorphine prescriptions filled by individuals who had no buprenorphine prescription in NYC during prior 3 years	· Adults and adolescents · Filled at least one prescription for buprenorphine · No buprenorphine prescription filled in NYC during prior 3 years	2006

^i^International Classification of Infectious Disease, 9^th^ revision (ICD-9).

^ii^ICD-9 codes specifying opioid misuse:

304.0 Opioid dependence

304.7 Combination opioid and any other drug dependence

305.5 Opioid abuse

965.0 Poisoning by opioids and related narcotics

E850.0 Accidental poisoning by heroin

E850.1 Accidental poisoning by methadone

E850.2 Accidental poisoning by opiates NEC

E935.0 Adverse effects in therapeutic use – heroin

^iii^ Opioids defined as 1) heroin or 2) prescription opioids: OxyContin, buprenorphine, non-prescribed methadone, or other opiate/synthetic.

^iv^ Substate-level data on opioid use is not published, but was obtained for New York City (NSDUH region A) from the Substance Abuse and Mental Health Services Administration (SAMHSA) for the purposes of this study.

^v^ The National Survey on Drug Use and Health (NSDUH) includes a series of questions to assess the prevalence of substance use disorders (i.e., dependence on or abuse of a substance) in the past 12 months. These questions are used to classify persons as dependent on or abusing specific substances based on criteria specified in the *Diagnostic and Statistical Manual of Mental Disorders*, 4th edition (DSM-IV) (American Psychiatric Association [APA], 1994). [http://www.oas.samhsa.gov/NSDUH/2k9NSDUH/2k9Results.htm#Ch7].

^vi^ Averages for calendar years 2005–2006 reported.

^vii^ Includes all non-hospital facilities of the New York City Department of Corrections providing methadone for detoxification or maintenance treatment: Eric M. Taylor Center, Anna M. Kross Center, Rose M. Singer Center.

^viii^ The DOHMH Bureau of Alcohol and Drug Use Prevention, Care and Treatment conducted an additional in-depth review of Medical Examiner records to define in further detail the substances involved and circumstances of overdose death. This data was previously reported (NYC DOHMH Vital Signs, February, 2010); only data from calendar year 2006 data was included in our analysis. Paone D, Heller D, Olson C, Kerker B. Illicit Drug Use in New York City. NYC Vital Signs. 2010;9(1):1–4. [http://www.nyc.gov/html/doh/downloads/pdf/survey/survey-2009drugod.pdf].

### *Description of the datasets*

Datasets containing detailed information on characteristics of the study population were classified as ‘primary.’ Those providing only general information about the population of interest (i.e. frequency of cases) were classified as ‘supplemental’ datasets.

### **Primary datasets**

*Statewide Planning and Research Cooperative System (SPARCS)* is an administrative reporting system of the New York State Department of Health (NYS DOH) that comprehensively records all hospital inpatient and outpatient visits to New York State hospitals [22]. Access to SPARCS data used in the current analysis was provided by the NYS DOH. The inpatient data records hospital discharges, while the outpatient data records emergency department (ED) and ambulatory surgery admissions. SPARCS data provides patient-level detail on demographic characteristics; medical procedures and principal, secondary and admitting diagnoses as classified by International Classification of Infectious Diseases, 9^th^ revision (ICD-9) diagnostic codes; services received; and charges for each treatment episode. Cases included in our analysis were drawn from the hospital discharge and ED admissions data. The ICD-9 diagnostic codes used to define ‘opioid use’ cases are listed in Table 1.

*Client Data System (CDS)* is an administrative data set of the New York State Office of Alcoholism and Substance Abuse Services (OASAS) of the NYS DOH, which provided the data for this study. The CDS tracks all admissions to licensed providers of medical detoxification and substance abuse treatment services, and records patient-level demographics and provides detailed information about recent substance use behaviors. Data is collected from each patient at the time of admission, and all information is based on client self-report. Cases were drawn from both the detoxification and substance abuse treatment program datasets, and limited to individuals reporting one or more opioids as a primary, secondary or tertiary drug of abuse. We did not distinguish between individuals who entered drug treatment voluntarily and those who were mandated to treatment by the criminal justice system through alternative to incarceration and similar programs.

### **Supplemental datasets**

*National Survey on Drug Use and Health (NSDUH)* is an annual household survey conducted by the U.S. Department of Health and Human Services, Substance Abuse and Mental Health Services Administration (SAMHSA). It is the primary source of information on the prevalence and patterns of substance use in the general population, age 12 and older [23]. Information collected includes patterns and severity of lifetime, past year and current drug use. Population prevalence is estimated from the survey sample using established NSDUH methodology [24]. Substate-level data on opioid use is not published, but was obtained from SAMHSA for New York City (NSDUH region A) by the NYC DOHMH (Bureau of Alcohol and Drug Use Prevention, Care and Treatment), which provided the data for this analysis. Cases included were limited to individuals classified as having abuse or dependence on heroin or prescription opioids.

The Bureau of *Correctional Health Services* in the NYC Department of Health and Mental Hygiene (NYC DOHMH) records on-site health services received by New York City jail inmates, including the number receiving methadone for detoxification or maintenance treatment of opioid dependence during the period of incarceration. Jail rather than prison inmates are included in this analysis because of their shorter length of stay. With an average length of stay of just 37 days [25], most of these inmates are expected to rejoin the community within the calendar year, thus adding to the population of New York City opioid users eligible for drug services. Data used in this study were provided by Correctional Health Services and do not allow identification of unique individuals.

The Bureau of *Vital Statistics* in the NYC DOHMH maintains records of unintentional drug overdose deaths. The Bureau of Alcohol and Drug Use Prevention, Care and Treatment conducted an additional review of Medical Examiner records for these cases to detail the substances involved and circumstances of overdose death. These publicly available overdose data were used in our analysis [6].

*Prescription records* are collected by the NYS DOH for all controlled substances prescribed and dispensed in New York State, including buprenorphine. Oral buprenorphine is indicated exclusively for treatment of opioid dependence, and may be prescribed by qualified physicians for maintenance or detoxification treatment. Data is collected on the total number of buprenorphine prescriptions filled, as well as the number of individuals filling a buprenorphine prescription for the first time. Data on buprenorphine prescriptions dispensed in New York City is regularly provided to the NYC DOHMH, Bureau of Alcohol and Drug Use Prevention, Care and Treatment, and the Bureau Assistant Commissioner (author DH) provided summary statistics for the current analysis. Buprenorphine treatment entrants were defined as those individuals filling a buprenorphine prescription in 2006 who did not fill a buprenorphine prescription within New York City in the prior three years.

Notably, we did not use data from the Drug Abuse Warning Network (DAWN). DAWN is a federal surveillance system tracking substance use-related ED visits based on chart reviews rather than ICD-9 codes, and is often applied to assessments of the health burden of illicit drug use at the local and national level [26]. We chose to use SPARCS instead of DAWN to track emergency department admissions in our analysis because DAWN data reflects a rate projection based on a sample of patient visits rather than an inclusive count of all ED admissions.

### **Analysis**

Three subpopulations of opioid users were defined. The general population (G) refers to opioid users identified through population health and criminal justice data. Drug services entrants (D) are those entering inpatient detoxification or drug treatment programs, or filling their first buprenorphine prescription. Medical services recipients (M) are those identified in the SPARCS datasets from inpatient hospitalizations (based on hospital discharge data) or emergency room admissions.

#### Estimation of subpopulations

We calculated the number of opioid users identified within each dataset according to the case definitions specified in Table 1. Within the primary datasets, cases were limited to unique individuals. In the SPARCS data, this was accomplished by creating a unique identifier based on SPARCS UPID, (an anonymous identifier provided in the datasets), in combination with date of birth and sex. In the CDS data, individuals were identified using a unique identifier constructed from sex, date of birth, last four digits of social security number, and first two characters of last name. To eliminate duplication, cases were further restricted to the first admission of the calendar year for each individual, both within and between the detoxification and substance abuse treatment program datasets. In the supplemental datasets, NSDUH methodology provided prevalence estimates [24]. Overdose deaths are unique by definition. The number of individuals receiving buprenorphine is tracked by the NYS DOH utilizing identifying information included on each filled prescription. We were not able to limit the correctional health services data to unique individuals.

The total number of individual opioid users captured in each subpopulation was then estimated. While we were able to limit cases within most datasets to unique individuals, we were not able to track individuals between the various data sources. This introduced the possibility of over-counting individuals who appeared in more than one data source. For example, an individual who had both an emergency room visit for medical reasons and a drug treatment program admission in 2006 may have been counted twice in our analysis, because one admission would be captured in the SPARCS data, and the other in the CDS. To account for this we incorporated ‘expansive’ and ‘restrictive’ estimates of the population size, and examined varying degrees of overlap between the datasets defining our subpopulations.

In estimating the ‘general population’ (G) of opioid users, the expansive estimate assumed no overlap between NSDUH and the corrections population or overdose decedents. The most restrictive estimate assumed that individuals identified through corrections or overdose deaths were already accounted for by the NSDUH sampling strategy and estimation method, even though corrections populations are not surveyed directly. In the ‘drug services population’ (D), the CDS dataset identified unique individuals admitted to either drug treatment or medical detoxification services. The only potential duplication of drug services recipients would thus be through double counting of those who received a prescription for buprenorphine in addition to detoxification or drug treatment services. Therefore, the more expansive estimate assumed that individuals initiating buprenorphine treatment and those entering detoxification or drug treatment services were distinct, whereas the more restrictive estimate assumed that buprenorphine recipients were already counted among the drug treatment admissions.

For the ‘medical services’ (M) population, any individuals who were hospitalized for drug detoxification or rehabilitation procedure(s) were identified using International Classification of Infectious Diseases, 9^th^ revision (ICD-9) diagnostic codes (ICD-9 codes 9461–9). These cases were removed from the analysis of the medical services population, because detoxification and rehabilitation admissions are comprehensively recorded in the CDS data. This step eliminated duplication of individuals appearing in both the CDS and SPARCS datasets. Unique individuals were identified within and across the SPARCS inpatient and outpatient data using our unique identifier.

#### Estimation of the total opioid user population

Adding the combined totals from each subpopulation provided an expansive and a restrictive unadjusted estimate of the opioid user population. However, combining these subpopulations introduced further potential for duplication of individuals who may appear in more than one group. To account for this, we examined two scenarios describing degrees of overlap between our general, medical services, and drug services subpopulations.

The expansive estimate assumed that there was no overlap between subpopulations. While some opioid users likely appear in more than one subpopulation, this approach may compensate for the fact that opioid users are a hidden population that is likely to be undercounted in all datasets [27,28]. The restrictive estimate assumed that the general population estimate accurately identifies all opioid users, thus eliminating the need to additionally include those individuals presenting for medical and drug services. The midpoint between the expansive and restrictive estimates was considered our best estimate of the total opioid user population.

#### Estimation of treatment entrants

We identified two subpopulations to formulate an estimate of the number of opioid users entering drug treatment in 2006. The first subpopulation came from the CDS data, which identifies those individuals citing opioid(s) as a primary, secondary, or tertiary drug of abuse upon entry to a treatment program. In this analysis of drug treatment entrants, individuals admitted for detoxification only were excluded, unless they also entered a treatment program in 2006. The second subpopulation was identified through buprenorphine prescription records. To account for potential double-counting of individuals receiving buprenorphine who may have also attended a drug treatment program, we calculated both expansive and restrictive estimates of the number of treatment entrants. The midpoint of the expansive and restrictive estimates was used as our estimate of the total number of treatment entrants.

#### Description of opioid users entering drug services and hospital-based medical services

After applying the specified case definitions to restrict the dataset to individual opioid users, we used descriptive statistics to examine the demographic and geographic characteristics of cases identified in the CDS (drug services) and SPARCS (hospital-based medical services) data. County of residence was identified by the individual’s home zip code. The drug services data included individuals admitted to drug treatment, detoxification services, or both during 2006. The hospital services data was divided into emergency room (ER) and inpatient admissions. In this descriptive analysis, opioid users who had hospitalizations for detoxification or rehabilitation were included in the hospital inpatient data, (along with those hospitalized for medical reasons), in order to provide a more comprehensive picture of the opioid user population accessing hospital-based acute medical care. This is in contrast to the prevalence estimations, in which individuals admitted for detoxification or rehabilitation were excluded from the SPARCS data to avoid duplication.

## **Results**

These data sources identified 126,681 cases of opioid use among New York City residents in 2006 (Table 2). Based on our case definitions, all of these opioid users have attributes consistent with opioid abuse, dependence, or poisoning, and would thus qualify for services to address their substance use [20,21].

**Table 2:** Opioid user subpopulations: New York City, 2006

**Subpopulation**	**Dataset**	**Opioid misusers (N)**	**Subpopulation estimation definitions**	**Subpopulation estimates (N)**	**Midpoint estimate (N)**
**General Population (G)**			Expansive = G_E_ = a + b + c Restrictive = G_R_ = a	G_E_ = 79,245 G_R_ = 59,680	69,463
a. Household survey	NSDUH	59,680			
b. NYC jail inmates receiving methadone	Correctional Health Services	18,958			
c. Overdose deaths involving opioids	Vital Statistics	607			
**Drug Services (D)**			Expansive = D_E_ = d + e Restrictive = D_R_ = d	D_E_ = 29,818 D_R_ = 26,938	28,378
d. Detox and drug treatment programs	CDS	26,938			
e. Buprenorphine	Prescription records	2,880			
**Medical Services (M)**			Total = M = f + g-h	M = 17,618	17,618
f. Hospital inpatient^i^	SPARCS	11,058			
g. Emergency room	SPARCS	7,856			
h. Both inpatient and emergency admissions^ii^	SPARCS	(−)1,296			
**UNADJUSTED TOTALS (T)**		126,681	Expansive = T_E_ = G_E_ + D_E_ + M Restrictive = T_R_ = G_R_ + D_R_ + M	T_E_ = 126,681 T_R_ = 104,236	115,459

^i^ Individuals hospitalized for medical reasons only; excludes those hospitalized only for drug/alcohol detoxification or rehabilitation.

^ii^ Individuals having both a medical hospitalization and an emergency room admission for an opioid-related diagnosis in calendar year 2006.

Opioid users were categorized into three subpopulations, of which the general population (G) was the largest, with a midpoint estimate of 69,463 individuals. The majority were identified from the NSDUH survey. The 607 unintentional opioid poisoning overdose decedents represented 0.9 % of this subpopulation. Entrants to drug services (D) were the second largest subpopulation, with an estimated 28,378 individuals. Ten percent (2,880) of this group received a prescription for buprenorphine. Medical service recipients (M) were the smallest of our three subpopulations, but still represented an estimated 17,618 opioid users.

**Figure 1:**
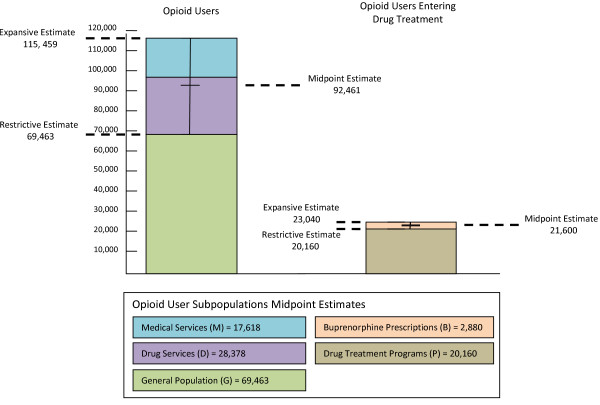
Estimation of total opioid users compared to opioid users entering drug treatment.

Adding the midpoint estimations for our three subpopulations produced an expansive estimate of 115,459 opioid users (Figure 1). The restrictive estimate, which assumed that all opioid users were represented in the general population estimate, lowered the estimation to 69,463. An estimated 21,600 entered drug treatment, either by enrolling in a substance abuse treatment program or initiating treatment with buprenorphine.

Characteristics of opioid users identified in the drug services versus hospital-based medical services data are shown in Table 3. Opioid users had a mean age of 40 years or greater, with hospital inpatients being considerably older. The majority were male, though there was greater representation of females in the hospitalized population than in drug treatment programs. The Bronx was the county with both the greatest number and proportion of opioid users. Bronx residents were also well represented in the drug services population, with a rate of 814 admissions per 100,000 adults.

**Table 3:** Characteristics of adult opioid users admitted to drug and medical services: NYC, 2006

**Characteristic**	**Drug Services Entrants (CDS)**				**Emergency Room Admissions (SPARCS)**				**Hospital Inpatient Discharges (SPARCS)**			
	Opioid users^a^ N (%)	Total individuals entering drug services^b^ N (%)	Opioid users in drug services population^b^ (%)	Opioid users in NYC(county) population^c^ (Rate/100,000)	Opioid users^a^ N (%)	Total individuals admitted to ER N (%)	Opioid users in ER population (%)	Opioid users in NYC(county) population^c^ (Rate/100,000)	Opioid users^a^ N (%)	Total individuals admitted to hospital^b^ N (%)	Opioid users in hospital population^b^ (%)	Opioid users in NYC(county) population^c^ (Rate/100,000)
Total	26,938 (100.0)	80,768 (100.0)	33.4	429	7,856 (100.0)	1,129,68 (100.0)	0.70	125	24,924 (100.0)	615,663 (100.0)	4.05	397
Age (years)												
Mean	41	40			41	42			44	52		
SD	9.5	11.0			10.1	17.3			10.6	20.9		
Median	40	40			41	39			44	51		
Range	18-76+	18-76+			18-116	18-124			18-99	18-116		
Sex												
Male	20,281	61,065	33.2	323	5,649	484,228	1.17	90	17,502	233,786	7.48	279
	(75.3)	(75.6)			(71.9)	(42.9)			(70.2)	(38.0)		
Female	6,657	19,703	33.8	106	2,207	645,353	0.34	35	7,422		1.94	118
	(24.7)	(24.4)			(28.1)	(57.1)			(29.8)	(62.0)		
County of Residence												
New York	6,917	21,501	32.2	517	2,068	222,181	0.93	155	6,439	122,459	5.26	482
	(25.7)	(26.6)			(26.3)	(19.7)			(25.8)	(19.9)		
Queens	3,607	13,328	27.1	206	795	246,088	0.32	45	3,066	145,271	2.11	175
	(13.4)	(16.5)			(10.1)	(21.8)			(12.3)	(23.6)		
Kings	7,085	21,288	33.3	382	2,253	333,495	0.68	121	6,544	190,454	3.44	353
	(26.3)	(26.4)			(28.7)	(29.5)			(26.3)	(30.9)		
Bronx	8,172	20,500	39.9	841	2,548	268,925	0.95	262	7,953	121,770	6.53	819
	(30.3)	(25.4)			(32.4)	(23.8)			(31.9)	(19.8)		
Richmond	1,157	4,151	27.9	319	192	58,919	0.33	53	922	35,709	2.58	254
	(4.3)	(5.1)			(2.4)	(5.2)			(3.7)	(5.8)		

^a^ Unique individuals; persons younger than 18 years are excluded.

^b^ Includes individuals who had admissions for detoxification or rehabilitation procedures.

^c^ Total NYC and county population measures represent the 5 NYC boroughs for ages 18 and older, 2006. Downloaded August 16, 2010 from NYC.gov epiquery function: https://a816-healthpsi.nyc.gov/epiquery/EpiQuery/Census/index2001.html.

We found that opioid users had high rates of acute care hospital utilization. Overall, they represented 4.1 % of all individuals hospitalized and 0.7 % of all individuals admitted to the emergency room in 2006. In total, opioid users had 44,154 hospitalizations, 20,959 (47 %) of which were for medical reasons (i.e. not for detoxification or rehabilitation). They incurred an additional 10,053 emergency room visits that did not result in hospitalization (data not shown).

## **Discussion**

Our analysis sought to provide a rigorous estimation of the opioid user population by grounding prevalence estimates in a range of datasets that are not often applied to this question. Although even this diversified approach may not capture the full scope of opioid use, it does allow construction of a more comprehensive picture of opioid users in New York City, including prevalence, service utilization, and demographic characteristics.

There is clearly a need for expanded drug services for opioid users in New York City. Even using our most restrictive estimations, there were over 69,000 opioid users residing in New York City in 2006. While over 21,000 individual opioid users did initiate treatment, this represents a small fraction of the identified opioid user population. Moreover, because close to half of treatment admissions are mandated each year in New York City, including one-third by criminal justice authorities (i.e. courts, probation, parole), one can assume a considerable portion of this fraction did not initiate treatment voluntarily.

The current drug treatment system alone cannot be relied upon to accommodate the needs of this population. Though maintenance treatment with an opioid agonist medication, (methadone or buprenorphine), is the best evidence-based treatment strategy for opioid dependence [29], not all of the opioid users identified here would necessarily qualify for, or accept, medication assisted treatment. Furthermore, accommodating these individuals within the existing substance abuse treatment system would involve doubling the capacity of New York City opiate agonist treatment programs, which currently serve approximately 37,500 patients [14]. Buprenorphine treatment in general medical settings provides an alternative means of treatment expansion, but slow uptake among physicians has thus far limited its reach.

While many opioid users would undoubtedly benefit from traditional drug treatment services, the availability of treatment slots is not the only barrier to engaging them in effective evidence-based care. Although these data sources largely capture the negative sequelae of opioid use (health problems, incarceration) that are more prominent among those with active substance use disorders, even non-dependent and early-stage users are vulnerable to drug-related problems, and may also have been identified here. Similarly, these data may include opioid users that were already enrolled in drug treatment, but nonetheless experienced problems related to drug use. Some opioid users could be served by less intensive treatment interventions, such as brief interventions or pharmacotherapy, provided in healthcare or community settings that are not traditionally considered part of the drug treatment system [30,31]. Many would benefit from harm reduction approaches to prevent the negative consequences of opioid use. A greater diversity, as well as a greater number, of treatment providers would thus be needed to deliver care to the full range of opioid users identified here. Public health authorities could apply this data to assess and orient the structural configuration of existing treatment services, and to expand engagement, access points, service types and modalities, and venues to better reach the sizeable under-served opioid user population.

Our approach does have a number of limitations. Most notably, we were unable to match individual opioid users across data sources. Capture-recapture analysis, which relies on identifying unique individuals across multiple datasets, can potentially improve the accuracy of prevalence estimation, and has been applied in municipalities outside the US to estimate populations of illicit drug users [32-35]. We chose not to use this approach because accessing fully identified individual-level data from multiple data sources was not feasible at the time of this analysis. Similarly, although it would be desirable to perform a validity check of the final estimates using an alternate method, this was beyond the scope of the present study. However, our findings do have face validity in light of prior estimates of the New York City opioid user population [1,13].

Our estimation of the medical services population relied on hospital data from inpatient and emergency room presentations, and did not capture individuals who utilized only ambulatory care services. Analysis of insurance claims data could contribute that information, but was not accessible for this analysis. Our data also fail to capture services provided within the Veteran’s Administration (VA) system.

There are several limitations inherent to the datasets themselves. Substance use is often not identified in medical settings [36-39] and substance use and other mental health diagnoses are poorly captured by medical administrative data [40-44]. Additionally, none of the datasets that rely on service utilization (drug treatment, detoxification, or hospital inpatient services) capture those with less problematic opioid use, who have not experienced the health and social sequelae of addiction that often drive users to seek care. As a result, we relied on the NSDUH for the general population estimation of opioid use, despite its known limitations [16,17]. Finally, our analysis is restricted to a single year-long period (2006), and thus does not capture trends in opioid use over time. This may be a particularly significant limitation with respect to prescription opioid use, which has been rising rapidly nationwide [9-11].

Despite these limitations, our study contributes to the understanding of the prevalence of opioid use in New York City by rigorously defining the dimensions and characteristics of the opioid user population. More sophisticated epidemiologic analyses of these multiple datasets, including capture-recapture and multiplier methods, could provide a more accurate estimate of the hidden population of opioid users who do not appear in any existing data sources. Our goal here was to take a first step toward that end by defining the number and range of individuals who met our case definition of problematic opioid use in health-related datasets.

## **Conclusion**

The health and social impact of opioid use is well recognized, but drug services have yet to adapt and expand to meet the needs of New York City’s sizeable out of treatment opioid user population. By providing a more comprehensive view of the opioid user population, our analysis could inform policy changes at the municipal and state level that would foster a broader spectrum of services for opioid users, and greater diversity of settings in which drug services are provided.

Efforts to reach this population could include provision of treatment and early intervention outside of specialized drug treatment programs, through greater involvement of the mainstream health care system. Though a minority of opioid users entered drug treatment in 2006, our analysis reveals that their contact with other aspects of the health system is routine. Many are utilizing hospital-based care, and many more are likely seen in ambulatory care settings, visits to which were not captured here. This presents an opportunity to expand both the scope and reach of drug services. Office-based treatment with buprenorphine, for example, offers the opportunity to reach individuals who do not typically seek care at substance abuse treatment programs. Greater adoption of screening and brief intervention (SBI) models is another potential avenue for expanding services to the large substance using population that is not actively seeking drug treatment.

These changes will occur only with strong public health leadership and targeted research that informs and supports changes within the complex systems of health care and drug treatment services. Buprenorphine’s potential has yet to be fully realized, in New York City and throughout the U.S., due to practitioner- and systems-level barriers [45-47]. There are important questions about how best to implement SBI, and its effectiveness for individuals with opioid abuse or dependence, but research can provide better models for identifying and engaging this population in health care settings [48]. Successful early intervention with these users could potentially prevent development of the severe sequelae of untreated opioid use disorders, including the health and social consequences that are starkly revealed here as statistics on hospitalizations, incarcerations, and overdose deaths.

## Abbreviations

Health surveys and datasets

CDS, Client Data System; DAWN, Drug Abuse Warning Network; ICD-9, International Classification of Infectious Diseases 9th revision; NSDUH, National Survey on Drug Use and Health; SPARCS, Statewide Planning and Research Cooperative System; NYC DOHMH, New York City Department of Health and Mental Hygiene; NYS DOH New, York State Department of Health; NYS OASAS, New York State Office of Alcoholism and Substance Abuse Services.

Agencies and Organizations

CDS, Client Data System; DAWN, Drug Abuse Warning Network; ICD-9, International Classification of Infectious Diseases 9th revision; NSDUH, National Survey on Drug Use and Health; SPARCS, Statewide Planning and Research Cooperative System; NYC DOHMH, New York City Department of Health and Mental Hygiene; NYS DOH New, York State Department of Health; NYS OASAS, New York State Office of Alcoholism and Substance Abuse Services.

## **Competing interests**

The authors have no conflicts of interest to disclose.

## **Authors’ contributions**

JM, DH and MG conceived of the study. JM directed the study and led the writing and analysis. DP, SW and SS contributed to the analyses. All authors advised on the analysis and contributed to the writing.

## **Authors’ information**

This study represents a collaborative effort between the NYU School of Medicine and the New York City Department of Health and Mental Hygiene, Bureau of Alcohol and Drug Use Prevention, Care and Treatment. At the time of writing, Daliah Heller, PhD, MPH was Assistant Commissioner of the Bureau, which has primary responsibility to develop and implement programs and policies to prevent the adverse health consequences of alcohol and drug use in New York City. The Bureau achieves its mission through developing and promoting policies, conducting and disseminating the results of epidemiologic analyses, promoting best practices in primary care and substance use service settings, overseeing contracted syringe exchange and substance use treatment services, and coordinating services across a wide range of governmental and community stakeholders. Denise Paone, EdD, BSN is the Bureau’s Director of Research and Program Development and Sharmila Shah, MD, MPH is Research Scientist.

## **Funding disclosure**

Dr. McNeely received support from NIH/NIDA (K23DA030395), NIH/NYU CTSI (KL2RR029891), and Cooperative Agreement Number T01 CD000146 from the Centers for Disease Control and Prevention (CDC). The contents of this paper are solely the responsibility of the authors and do not necessarily represent the official views of the New York City Department of Health and Mental Hygiene, the Centers for Disease Control, the National Institute on Drug Abuse or the National Institutes of Health.

## Pre-publication history

The pre-publication history for this paper can be accessed here:

http://www.biomedcentral.com/1471-2458/12/443/prepub
